# Metformin and sex: why suppression of aging may be harmful to young male mice

**DOI:** 10.18632/aging.100264

**Published:** 2010-12-30

**Authors:** Mikhail V. Blagosklonny

**Affiliations:** Department of Cell Stress Biology, Roswell Park Cancer Institute, Buffalo, NY, 14263, USA

Intriguingly, in this issue of *Aging*, Anisimov *et al* reported that lifelong treatment with metformin (an anti-diabetic drug with potentially anti-aging effects) was beneficial for female mice but shortened lifespan in males. Here I discuss why suppression of aging may be unfavorable in young males.

Metformin is used for treatment of type II (adult-onset) diabetes. Also, metformin and its analog phenformin prevent cancer and increase lifespan in rodents [1-7]. Yet, effects of metformin depend on mice strains and gender. In one strain (transgenic female HER-2/neu mice), metformin slowed down aging and tumor development [3]. In another strain (female SHR mice), metformin slowed down aging without inhibiting spontaneous tumorigenesis [4]. In third strain (female 129/Sv mice), metformin decreased carcinogenesis but only marginally increased life span [8]. Unexpectedly, in male mice of the same 129/Sv strain, metformin decreased the mean life span by 13% [8]. How can this be explained? There are 3 additional pieces to the puzzle. First, metformin via several mechanisms inhibits the mTOR pathway [9-15]. Second, inhibition of mTOR may explain the anti-aging effect of metformin [16,17]. Third, death rate was increased specifically in young males, thus decreasing their mean life span. Still, metformin did not affect lifespan of the last 10% of survivors and maximum life span [8].

## Death from “anti-aging”

Growth and aging share a common molecular mechanism [18]. Growth factors, insulin, cytokines, nutrients, and testosterone stimulate cellular growth in part by activating the mTOR pathway [19-30]. When a cell cannot grow in size, then activated mTOR contributes to senescent phenotype [31-33]. By promoting cellular aging, mTOR is involved in organismal aging and age-related diseases [34]. mTOR is essential earlier in life but also accelerates aging and age-related diseases (cancer). (Note: This is a clear-cut example of antagonistic pleiotropy [35]. As a matter of fact, any genetic pathway that accelerates aging must be beneficial earlier in life, otherwise it would be eliminated by, whatever weak, natural selection). Accelerated aging can be linked to vigor earlier in life [36,37]. In agreement, size and weight is associated with faster aging [38].

However, the degree of early-life benefits is slightly different in males and females. In the wild, young males have a higher risk of death (from accidents, competition and violence) than young females. (This is still the case in modern men and women). The higher death rate earlier in life, the more important is robustness. So males need to be stronger and bigger, to fight and compete and still survive. In many mammals (including 129/Sv mice and humans), males are larger. mTOR drives cellular growth and muscle hypertrophy [22,39-41], thus providing physical strength. Noteworthy, testosterone stimulates muscle cell hypertrophy via mTOR [39]. Even further, inhibitors of mTOR decrease testosterone levels in humans [42-44]. So it is reasonable to think that mTOR contributes to vigor of young males.

While decelerating aging, inhibition of mTOR may decrease robustness, tolerance to infections, cold temperatures and famine. This may be detrimental in unprotected environment. As discussed in detail [36], hypothetically, an anti-aging drug given to young men three centuries ago (when 75% of individuals died before the age of 26) would decrease lifespan due to death from infections, starvation and violence. This would preferentially eliminate weaker (and therefore slow-aging) individuals.

For laboratory mice, the environment is not completely protected because mice do not go to a doctor to treat infections, for instance. When environment is not completely protected, anti-aging treatment earlier in life can shift death from aging to death from external causes. Without repeating all arguments published recently [36,37], we can summarize: 1. Anti-aging agents may be harmful in young mice, when environment is not completely protected (external causes of death do exist). 2. This will affect males more than females. 3. In such conditions, this will preferentially eliminate weaker animals, who age slower. Robust, faster-aging animals will survive until aging. This effect will preclude extension of maximum lifespan, even if the aging is slowed in remaining alive (but faster aging) mice.

This may explain results by Anisimov et al published in this issue [8] and also may be applicable to other anti-aging modalities like calorie restriction (CR) and rapamycin. (Note: This may explain the lack of extension or even shortening lifespan by severe calorie restriction started early in life in some strains of inbred mice. If severe CR in young males leads to early death, then mostly fast-aging males would survive and then die relatively early too. Death of weak (slow-aging) males early in life may conceal potential anti-aging effects).

There may be other explanations. Metformin can cause side effects, which may be unrelated to its anti-aging effect and even unrelated to its “clinical” target AMPK. In humans, metformin can cause renaland gastrointestinal disturbances and other side effects. The challenge is to develop low doses of metformin (and especially their combinations with low doses of rapamycin) to suppress aging process without causing side effects. But even such modalities will not be probably indicated to healthy boys.

## References

[R1] Dilman VM, Berstein LM, Zabezhinski MA, Alexandrov VA, Bobrov JF, Pliss GB (1978). Inhibition of DMBA-induced carcinogenesis by phenformin in the mammary gland of rats. Arch Geschwul-stforsch.

[R2] Dilman VM, Anisimov VN (1980). Effect of treatment with phenformin, diphenylhydantoin or L-dopa on life span and tumour incidence in C3H/Sn mice. Gerontology.

[R3] Anisimov VN, Berstein LM, Egormin PA, Piskunova TS, Popovich IG, Zabezhinski MA, Kovalenko IG, Poroshina TE, Semenchenko AV, Provinciali M, Re F, Franceschi C (2005). Effect of metformin on life span and on the development of spontaneous mammary tumors in HER-2/neu transgenic mice. Exp Gerontol.

[R4] Anisimov VN, Berstein LM, Egormin PA, Piskunova TS, Popovich IG, Zabezhinski MA, Tyndyk ML, Yurova MV, Kovalenko IG, Poroshina TE, Semenchenko AV (2008). Metformin slows down aging and extends life span of female SHR mice. Cell Cycle.

[R5] Berstein LM (2009). Metformin, insulin, breast cancer and more. Future Oncol.

[R6] Anisimov VN (2010). Metformin for aging and cancer prevention. Aging (Albany NY).

[R7] Martin-Castillo B, Vazquez-Martin A, Oliveras-Ferraros C, Menendez JA Metformin and cancer: Doses, mechanisms and the dandelion and hormetic phenomena. Cell Cycle.

[R8] Anisimov VN, Piskunova TS, Popovich IG, Zabezhinski MA, Tyndyk ML, Egormin PA, Yurova MV, Rosenfeld SV, Semenchenko AV, Kovalenko IG, Poroshina TE, Berstein LM (2010). Gender differences in metformin effect on aging, life span and spontaneous tumorigenesis in 129/Sv mice. Aging.

[R9] Chan AY, Soltys CL, Young ME, Proud CG, Dyck JR (2004). Activation of AMP-activated protein kinase inhibits protein synthesis associated with hypertrophy in the cardiac myocyte. J Biol Chem.

[R10] Shaw RJ, Lamia KA, Vasquez D, Koo SH, Bardeesy N, Depinho RA, Montminy M, Cantley LC (2005). The kinase LKB1 mediates glucose homeostasis in liver and therapeutic effects of metformin. Science.

[R11] Tzatsos A, Kandror KV (2006). Nutrients suppress phospha-tidylinositol 3-kinase/Akt signaling via raptor-dependent mTOR-mediated insulin receptor substrate 1 phosphorylation. Mol Cell Biol.

[R12] Dowling RJ, Zakikhani M, Fantus IG, Pollak M, Sonenberg N (2007). Metformin inhibits mammalian target of rapamycin-dependent translation initiation in breast cancer cells. Cancer Res.

[R13] Mordier S, Iynedjian PB (2007). Activation of mammalian target of rapamycin complex 1 and insulin resistance induced by palmitate in hepatocytes. Biochem Biophys Res Commun.

[R14] Vazquez-Martin A, Oliveras-Ferraros C, Menendez JA (2009). The antidiabetic drug metformin suppresses HER2 (erbB-2) oncoprotein overexpression via inhibition of the mTOR effector p70S6K1 in human breast carcinoma cells. Cell Cycle.

[R15] Alimova IN, Liu B, Fan Z, Edgerton SM, Dillon T, Lind SE, Thor AD (2009). Metformin inhibits breast cancer cell growth, colony formation and induces cell cycle arrest in vitro. Cell Cycle.

[R16] Blagosklonny MV (2006). Aging and immortality: quasi-programmed senescence and its pharmacologic inhibition. Cell Cycle.

[R17] Blagosklonny MV (2007). An anti-aging drug today: from senescence-promoting genes to anti-aging pill. Drug Disc Today.

[R18] Blagosklonny MV, Hall MN (2009). Growth and Aging: a common molecular mechanism. Aging.

[R19] Schmelzle T, Hall MN (2000). TOR, a central controller of cell growth. Cell.

[R20] Rohde J, Heitman J, Cardenas ME (2001). The TOR kinases link nutrient sensing to cell growth. J Biol Chem.

[R21] Sarbassov DD, Ali SM, Sabatini DM (2005). Growing roles for the mTOR pathway. Curr Opin Cell Biol.

[R22] Wullschleger S, Loewith R, Hall MN (2006). TOR signaling in growth and metabolism. Cell.

[R23] Inoki K, Guan KL (2006). Complexity of the TOR signaling network. Trends Cell Biol.

[R24] Hands SL, Proud CG, Wyttenbach A (2009). mTOR's role in ageing: protein synthesis or autophagy?. Aging.

[R25] Edinger AL, Thompson CB (2002). Akt maintains cell size and survival by increasing mTOR-dependent nutrient uptake. Mol Biol Cell.

[R26] Huang J, Manning BD (2008). The TSC1-TSC2 complex: a molecular switchboard controlling cell growth. Biochem J.

[R27] Proud CG (2004). mTOR-mediated regulation of translation factors by amino acids. Biochem Biophys Res Commun.

[R28] Ma XM, Blenis J (2009). Molecular mechanisms of mTOR-mediated translational control. Nat Rev Mol Cell Biol.

[R29] Lee JH, Budanov AV, Park EJ, Birse R, Kim TE, Perkins GA, Ocorr K, Ellisman MH, Bodmer R, Bier E, Karin M (2010). Sestrin as a feedback inhibitor of TOR that prevents age-related pathologies. Science.

[R30] Dowling RJ, Topisirovic I, Alain T, Bidinosti M, Fonseca BD, Petroulakis E, Wang X, Larsson O, Selvaraj A, Liu Y, Kozma SC, Thomas G, Sonenberg N (2010). mTORC1-mediated cell proliferation, but not cell growth, controlled by the 4E-BPs. Science.

[R31] Demidenko ZN, Blagosklonny MV (2008). Growth stimulation leads to cellular senescence when the cell cycle is blocked. Cell Cycle.

[R32] Demidenko ZN, Zubova SG, Bukreeva EI, Pospelov VA, Pospelova TV, Blagosklonny MV (2009). Rapamycin decelerates cellular senescence. Cell Cycle.

[R33] Demidenko ZN, Blagosklonny MV (2009). Quantifying pharmacologic suppression of cellular senescence: prevention of cellular hypertrophy versus preservation of proliferative potential. Aging.

[R34] Blagosklonny MV (2010). Increasing healthy lifespan by suppressing aging in our lifetime: Preliminary proposal. Cell Cycle.

[R35] Blagosklonny MV (2010). Revisiting the antagonistic pleiotropy theory of aging: TOR-driven program and quasi-program. Cell Cycle.

[R36] Blagosklonny MV (2010). Why human lifespan is rapidly increasing: solving “longevity riddle” with “revealed-slow-aging” hypothesis. Aging.

[R37] Blagosklonny MV (2010). Why men age faster but reproduce longer than women: mTOR and evolutionary perspectives. Aging.

[R38] Anisimov VN, Ukraintseva SV, Yashin AI (2005). Cancer in rodents: does it tell us about cancer in humans?. Nat Rev Cancer.

[R39] Lee CH, Inoki K, Guan KL (2007). mTOR Pathway as a Target in Tissue Hypertrophy. Annu Rev Pharmacol Toxicol.

[R40] Wu Y, Bauman WA, Blitzer RD, Cardozo C (2010). Testosterone-induced hypertrophy of L6 myoblasts is dependent upon Erk and mTOR. Biochem Biophys Res Commun.

[R41] Altamirano F, Oyarce C, Silva P, Toyos M, Wilson C, Lavandero S, Uhlen P, Estrada M (2009). Testosterone induces cardiomyocyte hypertrophy through mammalian target of rapamycin complex 1 pathway. J Endocrinol.

[R42] Huyghe E, Zairi A, Nohra J, Kamar N, Plante P, Rostaing L (2007). Gonadal impact of target of rapamycin inhibitors (sirolimus and everolimus) in male patients: an overview. Transpl Int.

[R43] Skrzypek J, Krause W (2007). Azoospermia in a renal transplant recipient during sirolimus (rapamycin) treatment. Andrologia.

[R44] Lee S, Coco M, Greenstein SM, Schechner RS, Tellis VA, Glicklich DG (2005). The effect of sirolimus on sex hormone levels of male renal transplant recipients. Clin Transplant.

